# Increased Vaspin Levels Are Associated with Beneficial Metabolic Outcome Pre- and Post-Bariatric Surgery

**DOI:** 10.1371/journal.pone.0111002

**Published:** 2014-10-23

**Authors:** HuiLing Lu, Ponce Cedric Fouejeu Wamba, Marc Lapointe, Paul Poirier, Julie Martin, Marjorie Bastien, Katherine Cianflone

**Affiliations:** 1 Centre de Recherche de l’Institut Universitaire de Cardiologie & Pneumologie de Quebec, Université Laval, Québec, Canada; 2 Faculté Médecine, Université Laval, Québec, Canada; 3 Faculté de Pharmacie, Université Laval, Québec, Canada; 4 Department of Pediatrics, Tongji Hospital, HuaZhong University of Science and Technology, Wuhan Hubei, PR China; 5 Université de Yaoundé, Faculté des Sciences, Laboratoire de Nutrition et de Biochimie Nutritionnelle, Yaoundé, Cameroon; University of Bari Aldo Moro, Italy

## Abstract

**Purpose:**

Vaspin (visceral-adipose-tissue-derived-serine-protease-inhibitor) is a recently identified adipokine with putative insulin-sensitizing properties. Plasma vaspin responses to surgery-induced weight loss are sparse and contradictory.

**Design and Participants:**

We evaluated changes in vaspin levels and relationship to post-operative outcomes in men (n = 22) and women (n = 55) undergoing biliopancreatic-diversion/duodenal-switch bariatric surgery. Body composition and plasma parameters were measured at baseline, acutely (1 and 5 days) and medium-term (6 and 12 months) post-surgery.

**Results:**

Fasting preoperative vaspin concentrations were comparable in men vs women. The distribution was biphasic (both men and women) with a nadir of 2.5 ng/ml. Subjects were divided into high (≥2.5 ng/mL, HI-group) and low (<2.5 ng/mL, LO-group) vaspin level. Both groups had comparable sex distribution, age and BMI, but the HI-vaspin group had lower insulin, HOMA, and triglyceride and higher HDL-cholesterol, acylation stimulating protein (ASP) and IL-6 levels (all p<0.05). Post-operatively, both groups decreased BMI comparably over 12 months; the HI-vaspin group maintained high vaspin levels, while the LO-vaspin group gradually increased their levels with weight loss over 12 months. The HI-vaspin group maintained a better glucose, insulin, HOMA, fructosamine, HDL-cholesterol, and triglyceride profile throughout. The HI-vaspin group also had higher gamma-glutamyltransferase and ASP profiles. Finally, baseline vaspin level inversely correlated significantly with baseline and 12-month insulin, HOMA, triglyceride and positively correlated with HDL and ASP. Twelve-month vaspin also correlated similarly, including an inverse correlation with BMI.

**Conclusion:**

Globally, this study supports the concept of vaspin as a beneficial adipokine in obesity, which may potentially lead to possible therapeutic targets.

## Introduction

In addition to storing excess energy, adipose tissue secretes many bioactive peptides, termed “adipokines”, including leptin, adiponectin, acylation stimulating protein (ASP) and others that play important roles in metabolic homoeostasis through endocrine, paracrine, or autocrine pathways [1]. Over the past decade, the concept that the production of adipokines and pro-inflammatory molecules from hypertrophic/hyperplasic adipose tissue directly promote metabolic dysfunction, insulin resistance, type 2 diabetes progression and cardiovascular disease has become recognized [2]. Vaspin (visceral adipose tissue-derived serine protease inhibitor) is a newly identified adipokine with putative insulin-sensitizing properties, which has recently garnered interest. Vaspin was originally isolated from visceral adipose tissue in Otsuka Long-Evans Tokushima Fatty (OLETF) rats, a rodent model for insulin resistance and abdominal obesity, and was then found to be expressed in white adipose tissue of obese humans [3].

Administration of vaspin to obese mice improves glucose tolerance and insulin sensitivity [3]. When administered centrally, it reduces food intake leading to sustained reduction in blood glucose [3]. Further, in adipose tissue, vaspin directly exerts anti-inflammatory effects by inhibiting the expression of proinflammatory factors as resistin, leptin, and TNFα [4]. Recently, Phalitakul et al. demonstrated that vaspin inhibits TNFα-induced expression of ICAM-1, generation of reactive oxygen species and activation of NF-kB in vascular smooth muscle cells [5]. However, the exact mechanism of action is unknown, and the putative target protease of vaspin has not yet been identified [3].

Vaspin expression has been found in human adipose tissue, as well as skin, stomach, liver and pancreas [3]. While lean individuals had undetectable vaspin mRNA in both subcutaneous and visceral adipose tissue, the number of visceral adipose tissue samples that had detectable vaspin mRNA increased with increasing body mass [6]. However, the claim of predominant expression in visceral adipose tissue has been challenged [7]. Evidence in lean humans has demonstrated sexual dimorphism in vaspin serum levels, and increased vaspin concentration is associated with puberty, age, body mass index (BMI), leptin levels and insulin resistance [3]. However, these issues remain controversial, with several studies not demonstrating associations with gender, insulin sensitivity or obesity and fat distribution indices [8], [9], [10], [11]. By contrast, low serum vaspin is associated with myocardial ischemia, carotid stenosis, unstable angina, and coronary flow in a number of studies and the expression of vaspin in epicardial adipose tissue supports a potential role in cardiovascular disease (CVD) as well as in the atherosclerotic process [3]. Medications commonly used in patients with CVD have diverse effects with metformin decreasing [12] and atorvastatin increasing [13] circulating vaspin levels. Finally, the U-shaped relationship between vaspin levels and BMI recently reported [14], suggests that the regulation of serum vaspin is complex and may be regulated by unrecognized factors.

Changes in vaspin concentration with exercise and weight loss (exercise-induced, diet-induced and bariatric surgery induced) have been evaluated, yet the results are variable and interpretations remain inconsistent. Vaspin levels increase [14] or decrease [15] with 4 weeks exercise training, or remain unchanged [16] following exercise associated with a 10-month lifestyle modification. For example, Youn et al. [14] reported that 4 weeks of exercise training (60 mins, 3 days per week) significantly increased vaspin levels among normal individuals with glucose tolerance, impaired glucose tolerance and patients with type 2 diabetes. This same variability is also shown in weight loss studies [3]. With Mediterranean or low-carbohydrate diet-induced weight loss, vaspin exhibited cumulative decline despite partial weight regain during the timeline of the study [17]. By contrast, Koiou et al. did not observe any changes [18].

Plasma vaspin responses to bariatric surgery have also been controversial [19], [20], [21], which may partly be related to the type of bariatric surgery. Bariatric surgery is the most effective long-term treatment of severe obesity and is also very effective in the resolution of diabetes [22]. Resolution of diabetes often occurs even before weight loss [23], [22]. Rates of remission of type 2 diabetes have been reported from 57% up to 95%, depending on the surgical procedures which range from stomach restriction to intestinal malabsorption through biliopancreatic diversion [23], [22].

In the present study, we conducted a comprehensive investigation of short-term and medium-term endocrine and metabolic changes following biliopancreatic diversion with duodenal switch (BPD-DS) bariatric surgery in severely obese individuals. Our aims were to evaluate the acute and/or chronic changes in vaspin levels and the association with insulin sensitivity before and after weight loss, and the potential predictive contribution of pre-op vaspin levels to medium-term outcomes.

## Materials and Methods

### Study subjects

Subjects undergoing bariatric surgery (BPD-DS) were recruited at the Institut Universitaire de Cardiologie et de Pneumologie de Quebec (IUCPQ) affiliated with Laval University, Québec, Canada. Biliopancreatic diversion with duodenal switch surgery involves both restrictive and malabsorptive aspects. The restrictive portion involves removal of approximately 70% of the stomach along the greater curvature. The malabsorptive component involves rerouting a length of the small intestine, creating two separate pathways and one common channel. The experimental protocol was approved by the IUCPQ ethics committee and all patients gave their written informed consent. Subjects were selected in chronological order of surgeries, regardless of diabetic status or current medication, for participation based on the following inclusion criteria: patients (male and female) >18 years of age, BMI ≥40 or BMI ≥35 kg/m^2^ with associated comorbidities. Subjects who had previously undergone bariatric surgery or those bearing a pacemaker were excluded (patients with a pacemaker cannot undergo electrical bioimpedance assessment). Only subjects who completed the study (5 time points, with blood samples collected) were included for subsequent biochemical analysis. Laboratory procedures were completed before statistical analysis was performed.

### Anthropometric measurements

Subjects were assessed preoperatively and postoperatively (24 hours, 5 days, 6 months and 12 months). Blood samples were collected between July 2009 and May 2012. Height was measured using a stadiometer (SECA, 216 1814009, Brooklyn, NY, USA). Total body mass and BMI were evaluated by electrical bio-impedance balance (Tanita TBF-310, Tokyo, Japan) following a 12-hour fast. BMI was calculated as weight (kg)/height (m^2^). Medical history was collected for diabetes, hypertension, coronary artery disease and dyslipidemia as well as the corresponding pharmacological therapy. The information provided by the patient was confirmed by consulting clinical files.

### Blood collection and biochemical analysis

Venous blood was collected into EDTA containing tubes. Glycated hemoglobin (HbA1c) and fructosamine were evaluated in a fresh sample by turbidimetric inhibition immunoassay. All other tubes were rapidly placed on ice, centrifuged within minutes, plasma collected and frozen in aliquots at −80°C until analysis. Assays were measured in the hospital clinical biochemistry laboratory using standard methodology (fasting plasma glucose, cholesterol, triglyceride and HDL-cholesterol) or in the research laboratory (insulin, vaspin, ASP, IL-6 and ApoB). Plasma cholesterol, triglyceride (TG), HDL-cholesterol (HDL-C) and glucose were measured using colorimetric enzymatic kits (Cholesterol, TG and HDL-C: Roche Diagnostics Indianapolis, IN, USA; glucose: Wako Chemicals, Richmond, VA, USA). LDL-cholesterol concentration was calculated using the Friedewald formula [24]. Plasma insulin levels were measured by ELISA kit (Crystal Chem Inc, Downers Grove, IL, USA). Homeostatic model assessment of insulin resistance (HOMA-IR) was calculated from fasting plasma insulin and glucose levels as (insulin x glucose)/22.5, where the insulin concentration is reported as milliunits per liter and glucose as millimolar concentrations [25].

Vaspin was measured by commercial enzymatic immunoassay as per manufacturer’s instructions (Cedarlane, CA). The sensitivity was 1.85 ng/ml, with a 12% inter-assay coefficient of variation (CV) and a 5% intra-assay CV ASP was measured by sandwich ELISA assay as described, and data for ASP was reported elsewhere [26]. IL-6 was measured by ELISA immunoassay (R&D Systems, Ince, Minneapolis USA). Apolipoprotein B (ApoB) levels were measured by immunoturbidimetric method (Roche Diagnostics Integra 800 system).

### Statistical analysis

All results are expressed as mean ± standard deviation except where indicated as SEM. Values were compared using unpaired Student t-test or *X^2^* test as appropriate. Comparisons across different time periods were analyzed by repeated measures two-way ANOVA followed by Holm Sidak post-tests. As numerous variables were non-normally distributed, Spearman correlation analysis was used for all linear regression analyses. Graph Pad Prism (San Diego, CA, USA) and SigmaStat (San Rafaeal, CA, USA) software program was used for graph and statistical analysis. Statistical significance was set at p value <0.05, where p = NS indicates not statistically significant.

## Results

### Baseline fasting vaspin is biphasic

**Figure 1:**
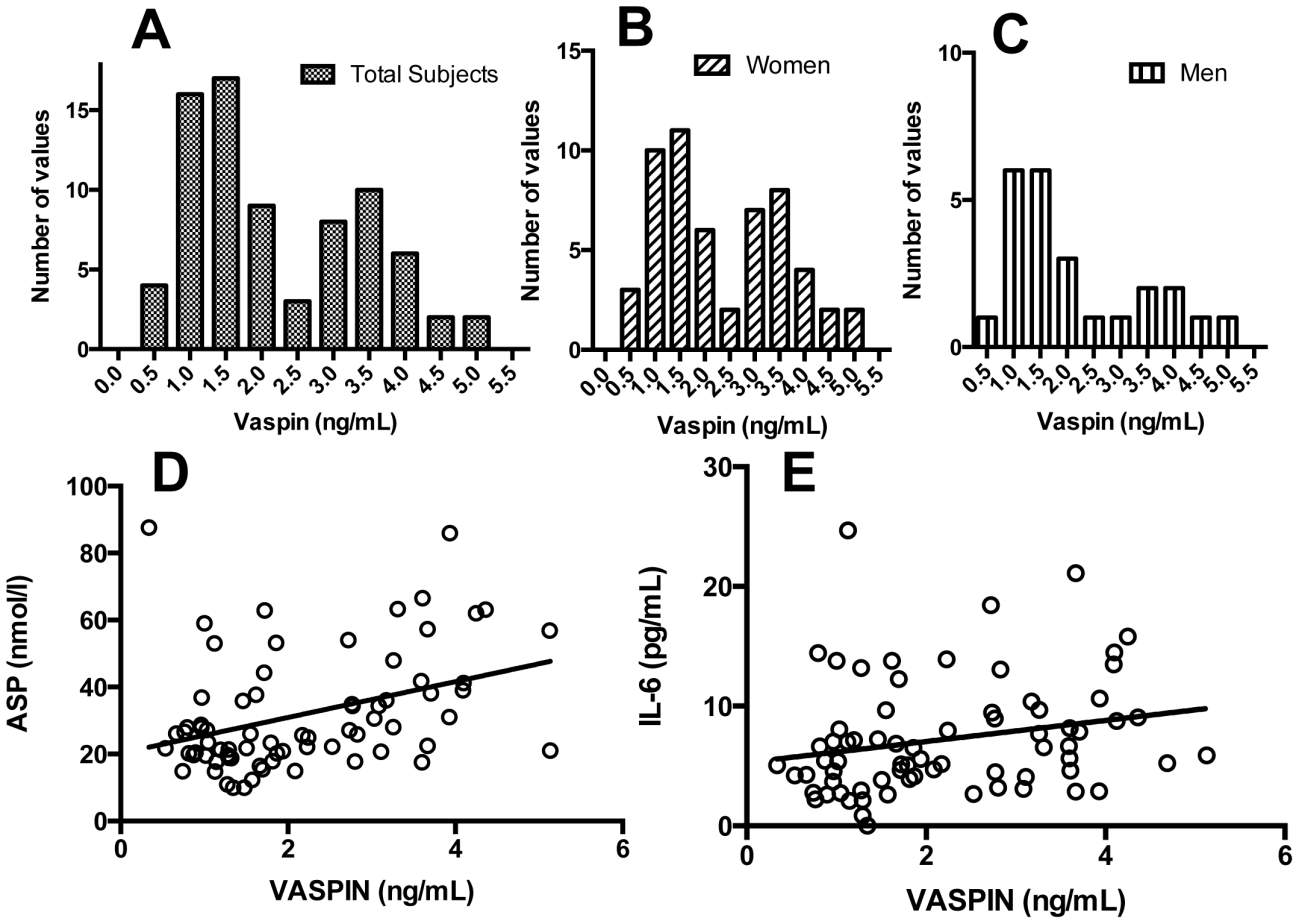
Preoperative Fasting Vaspin, Acylation Stimulating protein and IL-6 levels. Fasting vaspin (ng/mL), acylation stimulating protein (ASP, nmol/L) and interleukin-6 (IL-6, pg/mL) levels were measured in 77 severely obese patients (22 men and 55 women). Distribution of fasting plasma vaspin levels (ng/mL) according to frequency is given in all subjects (A), women (B) and men (C). D) Spearman correlation of fasting vaspin to fasting ASP (r = 0.371, p = 0.001). E) Spearman correlation of fasting vaspin to fasting IL-6 (r = .303, p = 0.01).

A total of 77 subjects (55 women and 22 men) underwent bariatric surgery with evaluation at 5 time points (baseline, 1 day, 5 days, 6 months and 12 months). Fasting plasma vaspin levels at baseline overall were 2.22±1.24 ng/mL and were no different between women (2.34±1.28) and men (1.96±1.1, p = NS). As shown in Figure 1A, distribution of fasting vaspin levels in all subjects depicted a biphasic profile. This was also true with analysis of women and men separately (Figures 1B and C); consequently all data presentations are with both sexes combined (as detailed below), unless otherwise indicated. Baseline vaspin levels correlated with a number of metabolic parameters including insulin, HOMA, plasma triglyceride and HDL cholesterol (Table 1). A number of other adipocytokines were also evaluated in this cohort [26], including acylation stimulating protein (ASP), IL-6 and visfatin. Both acylation stimulating protein and IL-6 levels correlated positively with vaspin (Figure 1D and E). All other parameters (as listed in Table 2) did not significantly correlate with baseline vaspin.

**Table 1:** Correlation of Vaspin with Parameters at Baseline and 12 months.

Vaspin Time/Parameter Time	Baseline Vaspin/Baseline r/p value	Baseline Vaspin/12-month r/p value	12-month Vaspin/12-month r/p value
BMI (kg/m2)		−0.197 (p<0.05)	−0.266 (p = 0.02)
Glucose (mmol/L)		−0.219 (p<0.05)	
Insulin (pmol/L)	−0.261 (p = 0.02)	−0.260 (p = 0.02)	−0.217 (p<0.05)
HOMA	−0.281 (p = 0.01)	−0.278 (p = 0.01)	−0.227 (p<0.05)
Triglyceride (mmol/L)	−0.209 (p<0.05)	−0.226 (p<0.05)	−0.228 (p<0.05)
HDL-cholesterol (mmol/L)	0.212 (p<0.05)	0.242 (p = 0.03)	
ASP (nmol/L)	0.371 (p = 0.001)	0.577 (p = 0.0001)	0.218 (p<0.05)
IL-6 (pg/mL)	0.338 (p = 0.003)		

Correlation of baseline and 12-month vaspin with baseline and 12-month parameters. All continuous variables listed in Table 2 were analysed. Only those with significant correlations are given.

**Table 2:** Baseline anthropometric and plasma parameters of high and low vaspin groups.

	LO VASPIN n = 46		HI VASPIN n = 31		
	AVE	SEM	AVE	SEM	P value
VASPIN	1.33	0.07	3.57	0.12	
Sex	16 M	30 F	6 M	25 F	ns
Age (years)	41.0	1.7	44.7	2.7	Ns
Height (m)	1.66	0.01	1.64	0.02	Ns
Weight (kg)	138	4	134	5	Ns
BMI (kg/m2)	49.7	1.1	49.9	1.2	Ns
%Fat mass	50.9	0.8	52.2	1.0	Ns
Diabetic Medication	16	(51%)	25	(54%)	Ns
Hyperlipidemic Medication	15	(49%)	22	(48%)	Ns
Glucose (mmol/L)	7.04	0.41	6.49	0.39	Ns
Insulin (pmol/L)	225	23	157	12	0.025
HOMA	10.6	1.3	6.8	0.7	0.031
HbA1c (%)	0.06	0.00	0.06	0.00	Ns
Fructosamine (umol/L)	221	6	210	5	Ns
Triglyceride (mmol/L)	1.87	0.18	1.44	0.13	0.078
Cholesterol (mmol/L)	4.67	0.15	4.63	0.14	Ns
LDL-cholesterol (mmol/L)	2.64	0.11	2.59	0.11	Ns
HDL-cholesterol (mmol/L)	1.21	0.04	1.39	0.06	0.012
Total Chol/HDL-C	4.01	0.18	3.49	0.15	0.039
Apolipoprotein B (g/L)	0.80	0.03	0.74	0.03	Ns
GGT (IU/L)	39.4	3.6	45.5	12.5	Ns
ALP (IU/L)	74.1	3.2	80.2	4.9	Ns
ALT (IU/L)	38.0	3.7	35.2	5.6	Ns
AST (IU/L)	28.8	2.8	26.9	2.9	Ns
ASP (nmol/L)	26.7	2.3	40.0	3.3	0.001
IL-6 (pg/mL)	6.42	0.71	13.92	4.20	0.044
Visfatin (ng/mL)	18.2	1.1	19.7	1.4	ns

Results are expressed as average ± sem, and compared by 2-mean t-test. Abbreviations: ALP: alkaline phosphatase; ALT: alanine transaminase; AST: asparatate transaminase; BMI: body mass index; GGT: gamma glutamyltransferase; HOMA: homeostatic model assessment; IL-6: interleukin-6; LDL: low-density lipoprotein; HDL: high-density lipoprotein.

Based on the biphasic profile of fasting vaspin levels, subjects were divided into 2 groups: 1) high vaspin group (HI Vaspin ≥2.5 ng/mL) and 2) a low vaspin group (LO Vaspin <2.5 ng/mL). The anthropometric and plasma profiles of both groups are given in Table 2. The HI vaspin group had lower insulin, HOMA, triglyceride, total cholesterol/HDL cholesterol ratio, but higher HDL cholesterol levels vs. the LO vaspin group. The HI vaspin group also had significantly higher plasma ASP and IL-6 levels (Table 2).

### High vs low baseline vaspin levels differentiates post-operative outcomes

**Figure 2:**
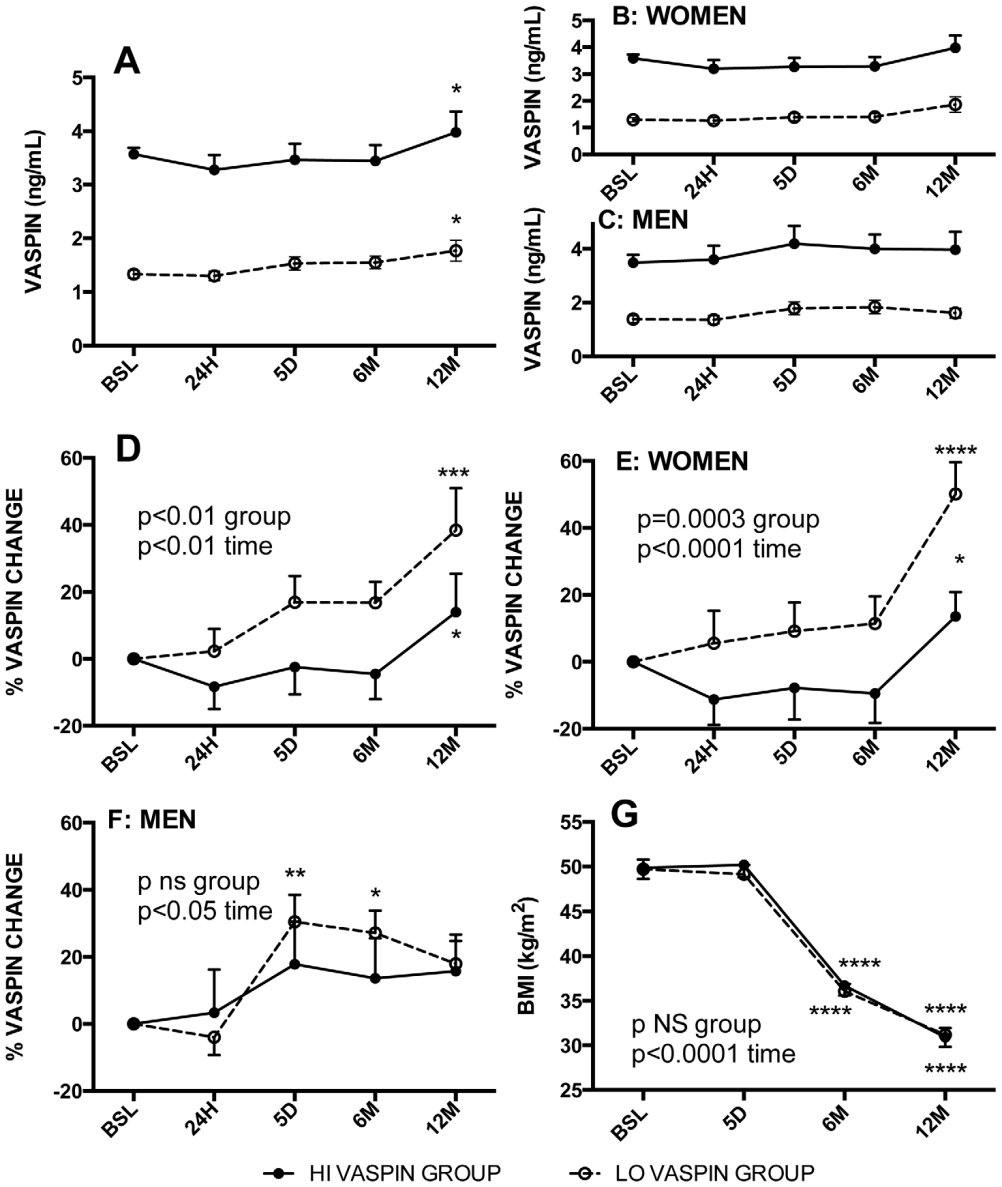
Preoperative and postoperative profile of fasting plasma vaspin and body mass index. Fasting plasma vaspin (ng/mL) and body mass index (BMI, kg/m^2^) were assessed at multiple time points: preoperative baseline (BSL) and postoperative 24 hours (24H), 5 days (5D), 6 and 12 months (6M and 12M). Patients were separated into 2 groups based on preoperative fasting vaspin levels: high vaspin (HI-grp, vaspin ≥2.5 ng/mL, black circles and low vaspin (LO-grp, vaspin <2.5 ng/mL, white circles). Pre- and post-operative fasting plasma vaspin in HI-grp and LO-grp in all subjects (A), women (B) and men (C). Percent change in post-operative vaspin levels relative to baseline (BSL) value in all subjects (D), women (E) and men (F). G) Pre- and post-operative BMI in HI-grp and LO-grp. Results are expressed as average ± sem, and were analyzed by 2-way ANOVA for time and group differences as indicated.

The baseline and post-operative vaspin profiles are shown in Figure 2A for all subjects combined. At all post-operative time points, average vaspin levels remained within their baseline assigned group. The profiles were comparable in both men and women (Figures 2B and C). However, over time, there was a gradual increase in fasting vaspin levels in both groups, with significant increases at 12 months in both groups (HI vaspin +14±%, p<0.05 and LO vaspin +38±%, p<0.001, Figure 2D). When separated into men and women, there were still increases in vaspin levels over time. In women (Figure 2E), the increase is greater in the LO vaspin group (while the HI vaspin increases at the last time point). In men, there are comparable percentage increases in both groups (group p ns) with significant increases over time (p<0.05)(Figure 2F). Overall the average increases range from 20% to 50%.

**Figure 3:**
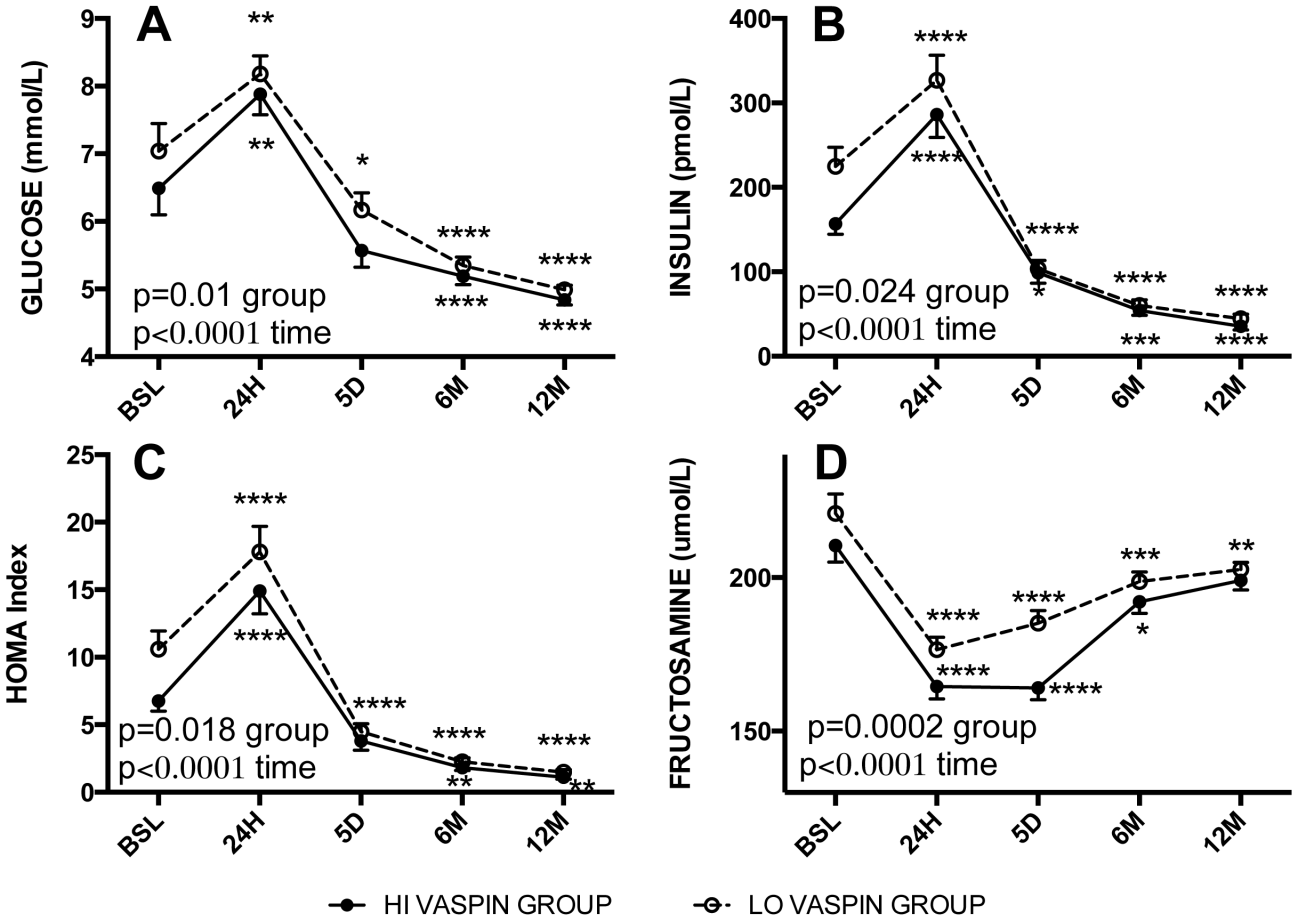
Preoperative and postoperative profile of fasting plasma glucose, insulin, HOMA and fructosamine. Fasting plasma glucose (A), insulin (B), HOMA (C) and fructosamine (D) were assessed at multiple time points: preoperative baseline (BSL) and postoperative 24 hours (24H), 5 days (5D), 6 and 12 months (6M and 12M). Patients were separated into 2 groups based on preoperative fasting vaspin levels: high vaspin (HI-grp, vaspin ≥2.5 ng/mL, black circles and low vaspin (LO-grp, vaspin <2.5 ng/mL, white circles). Results are expressed as average ± sem, and were analyzed by 2-way ANOVA for time (all p<0.0001) and group differences as indicated.

**Figure 4:**
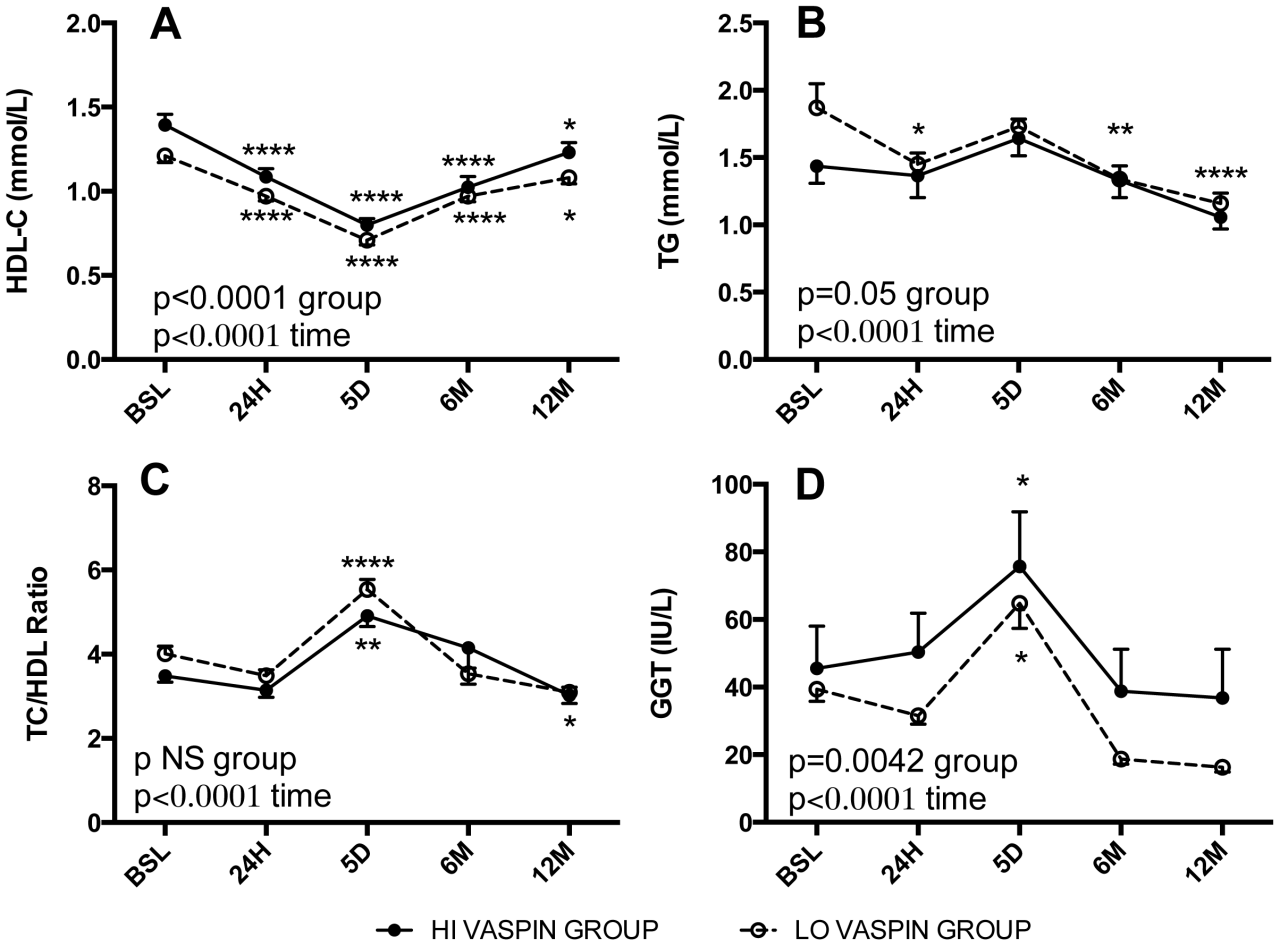
Preoperative and postoperative profile of fasting plasma HDL-cholesterol, triglyceride, total cholesterol/HDL-cholesterol ratio and gamma-glutamytransferase. Fasting plasma HDL cholesterol (HDL-C) (A), triglycerides (B), total cholesterol/HDL-cholesterol (TC/HDL ratio) (C) and gamma-glutamytransferase (GGT) (D) were assessed at multiple time points: preoperative baseline (BSL) and postoperative 24 hours (24H), 5 days (5D), 6 and 12 months (6M and 12M). Patients were separated into 2 groups based on preoperative fasting vaspin levels: high vaspin (HI-grp, vaspin ≥2.5 ng/mL, black circles and low vaspin (LO-grp, vaspin <2.5 ng/mL, white circles). Results are expressed as average ± sem, and were analyzed by 2-way ANOVA for time (all p<0.0001) and group differences as indicated.

The impact of high vs low pre-operative vaspin levels (HI vs. LO groups) on post-operative metabolic glucose, lipid and adipokine parameters was assessed. There was no difference in post-operative change in BMI (Figure 2G) for the group as a whole, or for men and women separately (Table 3). However, there were significant group differences in the glucose, insulin and HOMA profiles, with the HI vaspin group being lower throughout the one-year post-operative time period in all cases (Figure 3A–C, p = 0.04, p = 0.024 and p = 0.018, respectively). Further, fructosamine was also consistently lower in the HI vaspin group (Figure 3D, p = 0.0002). Similar profiles were demonstrated in both women and men (Table 3). Evaluation of lipid parameters indicate that the HI vaspin group had elevated HDL cholesterol (Figure 4A, p<0.0001) and lower plasma triglyceride (Figure 4B, p<0.05) throughout the post-operative period with no difference in total cholesterol, LDL cholesterol (not shown) or total cholesterol/HDL (Figure 4C). Similar profiles were demonstrated when divided into men and women separately (Table 3). The hepatic enzyme gamma-glutamyltransferase was significantly higher in the HI vaspin group throughout the one-year follow-up (Figure 4D, p = 0.004), in both men and women (Table 3), while there was no difference between HI-vaspin and LO-vaspin groups for the liver enzymes ALT, AST and ALP either at baseline or throughout the follow-up period (data not shown). This was also true when separated into groups of men and women (data not shown).

**Table 3:** Statistical comparison of HI Vaspin and LO Vaspin groups over time for all subjects as well as women and men separately.

Parameter	All Subjects	Women	Men
LO vs HI group	46/31	30/25	16/6
Vaspin % change	G<0.01;T<0.01	G = 0.005;T = 0.004	G NS; T<0.05
BMI (kg/m2)	G NS; T<0.0001	G NS; T<0.0001	G NS; T<0.0001
Glucose (mmol/L)	G<0.01; T<0.0001	G = 0.01; T<0.0001	G = 0.06; T<0.0001
Insulin (pmol/L)	G = 0.024; T<0.0001	G = 0.06; T<0.0001	G<0.05; T<0.0001
HOMA	G = 0.018; T<0.0001	G = 0.05; T<0.0001	G<0.05; T<0.0001
Fructosamine (umol/L)	G = 0.0002; T<0.0001	G = 0.0003; T<0.0001	G<0.05; T<0.0001
HDL-cholesterol (mmol/L)	G<0.0001; T<0.0001	G = 0.02; T<0.0001	G = 0.0002; T<0.0001
Triglyceride (mmol/L)	G<0.05; T<0.0001	G = 0.05; T = 0.0002	G = 0.06; T<0.05
T Chol/HDL-C	G NS; T<0.0001	G NS; T<0.0001	G NS; T<0.0001
GGT (IU/L)	G = 0.0042; T<0.0001	G = 0.009; T<0.0001	G<0.05; T<0.0001
ASP (nmol/L)	G<0.0001; T<0.0001	G = 0.006; T<0.0001	G = 0.002; T<0.0001
IL-6 (pg/mL)	G NS; T<0.0001	G NS; T<0.0001	G NS; T<0.0001
Visfatin	G NS; T NS	G = 0.008; T NS	G<0.05; T NS

Spearman correlation coefficients and p value for correlation between vaspin (baseline or 12 months) and various parameters (baseline and 12-month value). All parameters as given in Table 2 were tested. Only those with significance are presented.

**Figure 5:**
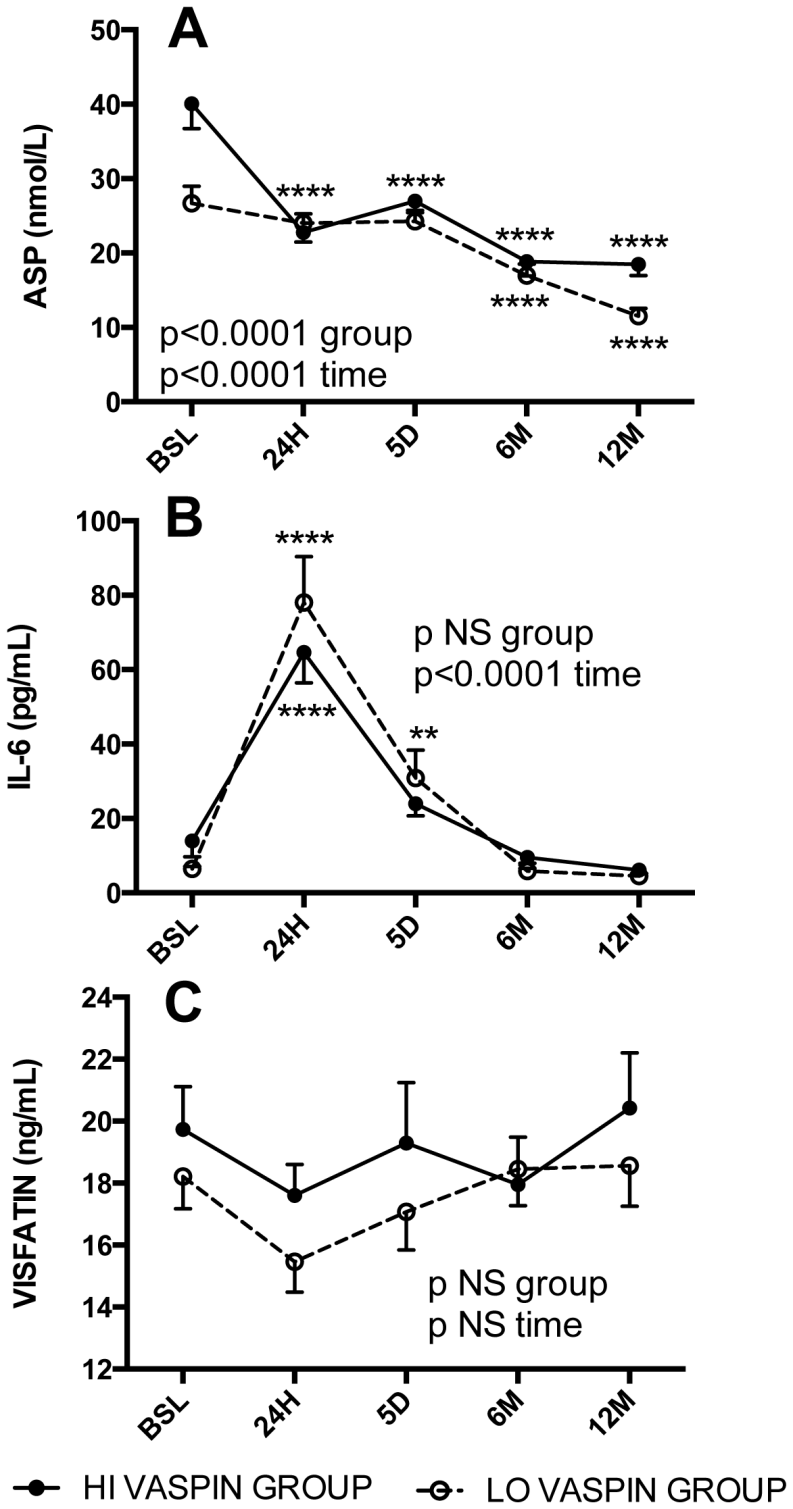
Preoperative and postoperative profile of fasting plasma Acylation Stimulating Protein, Interleukin-6 and Visfatin. Fasting plasma Acylation Stimulating Protein (ASP) (A), Interleukin-6 (IL-6) (B), and Visfatin (C) were assessed at multiple time points: preoperative baseline (BSL) and postoperative 24 hours (24H), 5 days (5D), 6 and 12 months (6M and 12M). Patients were separated into 2 groups based on preoperative fasting vaspin levels: high vaspin (HI-grp, vaspin ≥2.5 ng/mL, black circles and low vaspin (LO-grp, vaspin <2.5 ng/mL, white circles). Results are expressed as average ± sem, and were analyzed by 2-way ANOVA for time and group differences as indicated.

The relationship between HI vs LO vaspin groups and the adipokines ASP, IL-6 and visfatin levels are shown in Figure 5, where ASP was significantly higher in the HI vaspin group throughout the post-operative period (Figure 5A, p<0.0001). Similar profiles were obtained for all three adipocytokines when separated in to men and women groups (Table 3). Further, using forward stepwise regression, 26.6% of baseline vaspin levels could be predicted from a combination of fasting baseline ASP+visfatin levels (r = 0.515, p<0.001).

Since baseline vaspin levels correlated with both fasting baseline parameters (Table 1) as well as with post-operative changes over the 5 time points based on the two groups, the correlation between baseline vaspin levels and final outcome (12 months) as well as between 12-month vaspin levels and final outcomes were also evaluated. As shown in Table 1, baseline vaspin levels correlated negatively with final 12-month values of glucose, insulin, HOMA, plasma triglyceride and positively with HDL, indicating that higher vaspin levels are associated with a better metabolic outcome at 12 months. This is also true for post-operative weight, with higher vaspin levels associated with lower BMI. Further, as vaspin increased over the post-operative period, and more so in the LO vaspin group (Figure 2D), final (12 month) vaspin levels also correlated inversely with final values of insulin, HOMA, and triglyceride, as well as BMI, again indicating that higher levels of vaspin at 12 months are more strongly associated with a better metabolic outcome.

## Discussion

The prominent features of the current study are (i) a biphasic profile of vaspin levels in severely obese subjects in which vaspin correlates with glucose homeostasis, lipid parameters and adipokines, (ii) improved pre- and post-operative metabolic profile in patients with high pre-operative vaspin levels, (iii) an increase in vaspin levels post-operatively, specifically in patients with low pre-operative vaspin concentration, and (iv) at 12 months post-surgery, associations of high vaspin levels with lower insulin, HOMA, BMI and triglycerides.

A number of controversial and contradictory data have been reported regarding vaspin levels with respect to influence of sex dimorphism, age, obesity/fat distribution, and insulin resistance [3]. It has previously been suggested that vaspin levels are different between men and women, although this is controversial [3]. In the present study, while we noted a biphasic profile in vaspin levels, this response was not sex, age or obesity dependent. However, it has been suggested that sex dichotomy is lost in the presence of obesity [3], which is consistent with our data.

Vaspin levels in relation to obesity and fat distribution are controversial and correlations appear to be abrogated in patients with chronic disease [3]. While the present study consists primarily of severely obese individuals (BMI 35.9 to 75.5 kg/m^2^ pre-operatively and 23.1 to 53.3 kg/m^2^ post-operatively), nonetheless inverse correlations between pre-operative vaspin levels and 12-month vaspin levels with post-operative weight and BMI were observed.

Further, the association between fasting vaspin levels and insulin resistance markers (glucose, insulin and HOMA) is also controversial [3]. In the current study, this association was one of the strongest noted, and was present both pre-operatively as well as post-operatively (following weight loss). Overall, higher vaspin levels pre-operatively were associated with a better metabolic profile pre-surgery, as well as better metabolic outcome post-surgery. Interestingly, this was noted even at 1-day post-surgery, with differences in glucose, insulin, HOMA, and GGT as well as at 5-days post-surgery (glucose, GGT), even before any significant weight loss occurred. Further, at 12 months post-surgery, we documented inverse correlations, as patients with the highest vaspin levels (either pre-operative or at 12 months) had a better metabolic profile.

There are relatively few studies on vaspin changes in relation to weight loss. Exercise can result in increases, no change or decreases in vaspin levels [14], [15], [16]. Other studies have noted differing response to either diet-induced, or short-term-starvation weight loss [17], [18]. There are only 2 studies that have evaluated vaspin changes post bariatric surgery. Golpaie et al [19] evaluated vaspin changes after 6 weeks post-laparoscopic restrictive bariatric surgery (LRBS), demonstrating decreased vaspin levels (−25%) and BMI, without changes in insulin or glucose levels in a group of obese men and women. Handisurya et al [20] reported a decrease in vaspin levels at 12 months following Roux-en-Y gastric bypass (RYGB). In the present study, following BPD-DS, overall, vaspin levels increased post-operatively. Patients with lower vaspin levels to begin with, increased more over the 12-month period than the patients with high preoperative vaspin levels. While we can only speculate, perhaps the post-operative associations are dependent on the type of bariatric surgery (restrictive vs. malabsorptive). Further, BPD/DS is a complex surgery, and post-operatively, gastric restriction, lipid malabsorption, bile flux changes, altered entero-hepatic circuits, and changes in intestinal microflora are all potential mechanisms that may affect vaspin and all other parameters. However, the changes in vaspin levels did not occur early on (1-day and 5-days), but only occurred within the later time points (6 and 12 months) associated with weight loss, and at 12 months, there was an inverse correlation of vaspin with BMI. Within both groups there was variability, where some subjects had greater increases than others both in the HI vaspin and in the LO vaspin group. This inverse correlation would suggest that those subjects that had the greatest increases in vaspin, with the highest final (one year) values also reached the lowest BMI. This contrasts with the early changes in insulin noted here, and in other adipokines noted elsewhere such as ASP [26] and leptin [27], [28]. On the other hand, the weight-associated changes in vaspin levels are similar to post-operative changes noted in adiponectin levels [27], [28].

Globally, our results suggest that “higher is better” at least with respect to severely obese patients pre-and post- bariatric surgery. Further, in transgenic vaspin mice or following acute vaspin administration, increased vaspin levels resulted in improved glucose and insulin tolerance without changing adiponectin concentrations [29]. This is consistent with the view of vaspin as a “beneficial serpin in obesity and diabetes” [30]. We speculate that the contradictory changes in vaspin levels may be related to targeted mechanisms of action (vaspin-mediated vs vaspin-independent). For example, a treatment that directly increases vaspin production, will lead to increased vaspin action and improved metabolic parameters (vaspin-mediated compensation). On the other hand, a treatment that improves metabolic parameters without invoking vaspin action, would then result in decreased vaspin levels as it is no longer needed (decompensation).

Recent evidence suggests that vaspin-mediated functions may target multiple mechanisms and several target tissues. For example, it has been suggested that vaspin, as a serine protease, may block protease action, leading to less degradation of target hormone [3]. A recent publication demonstrates that vaspin inhibits kallikrein 7, and vaspin treatment of pancreatic islets leads to increased glucose-mediated insulin concentrations, effects lost using vaspin mutants [31]. However vaspin also acts through the cell surface receptors GRP78 [29], [32], leading to changes in Akt/PI3kinase signalling [30], pathways common to both insulin and ASP [33] targeting. These pathways may be involved in the vaspin effects on adipocytes decreasing leptin, TNFα and resistin, and increasing adiponectin and Glut4 [34], [35]. Effects of vaspin also extend to other physiological processes including food intake and inflammation with targets in brain, muscle, and endothelial cells among others [34], [35].

The limitations of the study are that all patients were severely obese which limits the generalizability of the data. Further, although the subjects were followed over time, the analyses remain correlational.

In conclusion, this study demonstrates associations of high vaspin levels with glucose and lipid-related metabolic parameters in severely obese patients, as well as post-operative increases in vaspin levels, and correlations of higher pre- and post-operative vaspin levels with better metabolic profile and lower BMI. Globally, this study supports the concept of vaspin as a beneficial serpin in obesity, which may potentially lead to possible therapeutic targets [34].
